# The Passing of Quarantine

**Published:** 1905-12

**Authors:** 


TEXAS MEDICAL JOURNAL.

AUSTIN, TEXAS.

A MONTHLY JOURNAL OF MEDICINE AND SURGERY.

EDITED AND PUBLISHED BY
F. E. DANIEL, M. D.
Mrs. F. E. DANIEL, Business Manager.

Published Monthly at Austin, Texas. Subscription price $1.00 a year in advance

### THE PASSING OF QUARANTINE.

I predict that the South has seen the last of the absurd and ruinous "quarantine" that totally arrests travel and traffic and paralyzes all commerce; that relict of barbarism so ruinous to the whole country.

In the early days, days of ignorance and panic, an infected vessel was held forty days, or until the infection should run out, the entire crew having the disease. Hence, "quarantine" (from *quarante,* forty.) Experience demonstrated that the period of detention could sometimes be shortened, until we got to speaking of a ten-day "quarantine" or a five-day "quarantine," a solecism and an absurdity; and it meant a total cessation of intercourse, enforced even at the muzzle of a loaded gun. There seemed to be no other course, as it was generally believed that yellow fever was "catching," and that it was carried in goods and merchandise and in the clothing of persons. Science has now dispelled this illusion, and shown that the disease is not "catching," and can be conveyed from one person to another only by the mosquito, and that in travel the only danger besides the presence in a car or ship of infected mosquitos, is that some passenger may have been bitten by an infected mosquito before starting on the trip and thus have the disease germinating in his blood. It is therefore possible, and only necessary, to guard against these two contingencies, to insure safety in intercourse with an infected locality; the first, by screening all cars and destroying by fumigation any mosquitoes that may possibly be lurking therein; the second, by inspection before embarking, and *en route,* of all passengers, and the detention somewhere on the line, of all suspected persons, long enough to show clearly whether or not the disease is incubating in any one; usually about five days. We can now understand why yellow fever disappeared from the North. Mosquitoes were killed.

Thus travel and interstate commerce can now go on uninterrupted; and now that the entire South with the exception of Mississippi, has expressed itself in favor of Federal control of epidemics (see resolution published elsewhere in this issue) no doubt that Congress and the legislatures will make suitable laws under which this sensible action can be made operative. Should the South be again invaded, I look to see the infection promptly exterminated, and inspection and fumigation, and screens and detention take the place of that ruinous quarantine which heretofore so effectually locked the wheels of travel and commerce, and led to such financial loss, worry and inconvenience. Inspection and detention of persons when necessary long ago took the place of ship quarantine in England and other parts of the world. It has been slow in reaching this country, but it has come at last, and is here to stay. The past season it was found effective in keeping yellow fever out of Texas, and confidence abides with our people now. Before, they, while having every confidence in the wisdom of our quarantine officer—had none in any other method of dealing with infection than by non-intercourse, and they expected him to enforce it and he did enforce it. It required some moral courage on Dr. Tabor's part to permit passenger trains and freight trains to enter Texas; but the result has shown the wisdom of his course. As it was, the expenses exceeded his available resources about $25,000, and there has been created a deficiency which the next Legislature must provide for. Had the fever gained entrance and a foothold in Texas where would the little "quarantine" appropriation of $45,000 have been? Swallowed up quicker, and we should have had to call on the M. H. S. as we did in 1882 when yellow fever broke out near Brownsville, and in 1894 to suppress the small pox epidemic at Eagle Pass. The latter cost the M. H. S. $214,000, as well as I remember.

It will be wise, therefore, to let the M. H. S. take entire charge, thus relieving the States of the enormous expense, and enabling the State health officer to apply the niggardly sum usually provided by Texas for quarantine to the prevention of diseases other than those of foreign origin. State's rights be d—hanged! We had a little argument on the subject of State rights along about 1861 to '65 and lost out. In fact I am much inclined to a centralization of power in the Federal Government; certainly a National Board of Health, and I am not so sure that I am not an Imperialist.

*Vale, Quarantine!*

